# Interaction of the Transcription Start Site Core Region and Transcription Factor YY1 Determine Ascorbate Transporter SVCT2 Exon 1a Promoter Activity

**DOI:** 10.1371/journal.pone.0035746

**Published:** 2012-04-20

**Authors:** Huan Qiao, James M. May

**Affiliations:** Department of Medicine, Vanderbilt University School of Medicine, Nashville, Tennessee, United States of America; University of Insubria, Italy

## Abstract

Transcription of the ascorbate transporter, SVCT2, is driven by two distinct promoters in exon 1 of the transporter sequence. The exon 1a promoter lacks a classical transcription start site and little is known about regulation of promoter activity in the transcription start site core (TSSC) region. Here we present evidence that the TSSC binds the multifunctional initiator-binding protein YY1. Electrophoresis shift assays using YY1 antibody showed that YY1 is present as one of two major complexes that specifically bind to the TSSC. The other complex contains the transcription factor NF-Y. Mutations in the TSSC that decreased YY1 binding also impaired the exon 1a promoter activity despite the presence of an upstream activating NF-Y/USF complex, suggesting that YY1 is involved in the regulation of the exon 1a transcription. Furthermore, YY1 interaction with NF-Y and/or USF synergistically enhanced the exon 1a promoter activity in transient transfections and co-activator p300 enhanced their synergistic activation. We propose that the TSSC plays a vital role in the exon 1a transcription and that this function is partially carried out by the transcription factor YY1. Moreover, co-activator p300 might be able to synergistically enhance the TSSC function via a “bridge” mechanism with upstream sequences.

## Introduction

Since most mammalian cells and all human cells are unable to synthesize vitamin C, or ascorbic acid, they are dependent upon uptake of the vitamin from their surroundings. This uptake is mediated primarily by one of two sodium-and energy-dependent vitamin C transporters, termed SVCT1(slc23a1) and SVCT2 (slc23a2) [1]. The SVCT1 is located primarily in intestinal epithelium and renal proximal tubule cells, where it mediates ascorbate absorption and reabsorption, respectively. The SVCT2, on the other hand, has a more generalized tissue distribution in most major organs, with highest expression noted in brain and neuroendocrine tissues, such as pituitary and adrenal gland.

The SVCT2 is crucial for ascorbate uptake in metabolically active and specialized tissues. Although SVCT2-deficient embryos typically survive until birth, they die shortly thereafter, failing to take a first breath and inflate the lungs [2]. The cause of death seems to relate to damage in the brain due to capillary hemorrhage. This is most evident in the cortex, but also occurs in areas of the lower brain crucial for control of body functions, including respiration [3].

In nucleated cells a variety of agents enhance SVCT2 expression at the levels of mRNA, protein, and function. In some instances this accompanies cell differentiation, such as with zinc [4], calcium/phosphate ions [5] and phorbol ester [6]. In others it is not related to cell differentiation, such as when induced by glucocorticoids [7], epidermal growth factor [8], or hydrogen peroxide [9]. Whereas these results show transcriptional regulation of the SVCT2, they do not define the molecular mechanism by which this occurs.

Concerning human SVCT2 regulatory regions, Rubin and co-workers identified two distinct promoters (CpG-poor exon 1a promoter and CpG-rich exon 1b promoter) located immediately upstream of the first two exons (termed exon 1a and exon 1b) [10]. The SVCT2 exon 1b is ubiquitously expressed in human and mouse tissues. Although this promoter doesn't contain a classical TATA box, it contains a functional initiator that binds Yin Yang-1 (YY1) and interacts with upstream Sp1/Sp3 elements in the proximal promoter region [10], [11]. These elements play a critical role in regulating YY1-mediated transcription of the exon 1b. Formation of YY1/Sp complexes on this promoter is required for its optimal function. Additionally, both EGR-1 and -2 were also detected in the protein complexes that bound the three GC boxes bearing overlapping binding sites for EGR/WT1 and Sp1/3. The EGR family factors, WT1 and MAZ were found to differentially regulate the exon 1b promoter activity [11].

In contrast to the ubiquitously expressed SVCT2 exon 1b, the expression of the SVCT2 exon 1a exhibits cell-specificity, found in some cell types and not in others [12]. Exon 1a is regulated by the interaction of the transcription factors Upstream Stimulating Factor (USF) and Nuclear Factor-Y (NF-Y), in that USF1/2 and NF-Y bind to the upstream sequence of the exon 1a promoter in a cooperativity-dependent manner and form an activating complex [12]. The formation of this NF-Y/USF complex is absolutely required for the full activity of the exon 1a promoter. Further, bisulfite genomic sequencing revealed that CpG methylation at the upstream USF-binding site predicted the observed cell-specific expression of this promoter. Specific methylation of this CpG site impaired both USF binding and the formation of the functional NF-Y/USF activating complex with a resulting decrease in promoter activity. Although these studies describe one mechanism of upstream regulation of the exon 1a promoter, it is also likely that activity of this promoter also depends on transcription factors binding on or near the transcription start site.

Due to the presence of ubiquitous transcription factors, the exon 1a 5′ region could be protected against remethylation in transient transfection and the exon 1a promoter clearly exhibited similar transcriptional activity in both exon 1a-expressing cells and non-expressing cells [12]. Thus, we employed both exon 1a-expressing cells (EA.hy926) and non-expressing cells (HeLa) further to characterize the sequences that comprise the transcription start site core (TSSC) of the SVCT2 exon 1a, which is positioned downstream of the E/Y boxes and overlaps the transcription initiation site. We determined that two protein-DNA complexes, TSSC(A) and TSSC(B), bind specifically to the TSSC region and identified TSSC(A) and TSSC(B) as YY1 and NF-Y, respectively. We also found that the YY1 protein and its binding site are necessary for the full promoter activity and that YY1 activates transcription from the exon 1a promoter. Additionally, the non-DNA-binding transcriptional co-activator p300 is also involved in the transcriptional regulation of the SVCT2 exon 1a, very likely via a “bridge” mechanism.

## Results

### The TSSC of the exon 1a promoter contains two adjacent YY1 binding sites and binds two protein complexes

**Figure 1:**
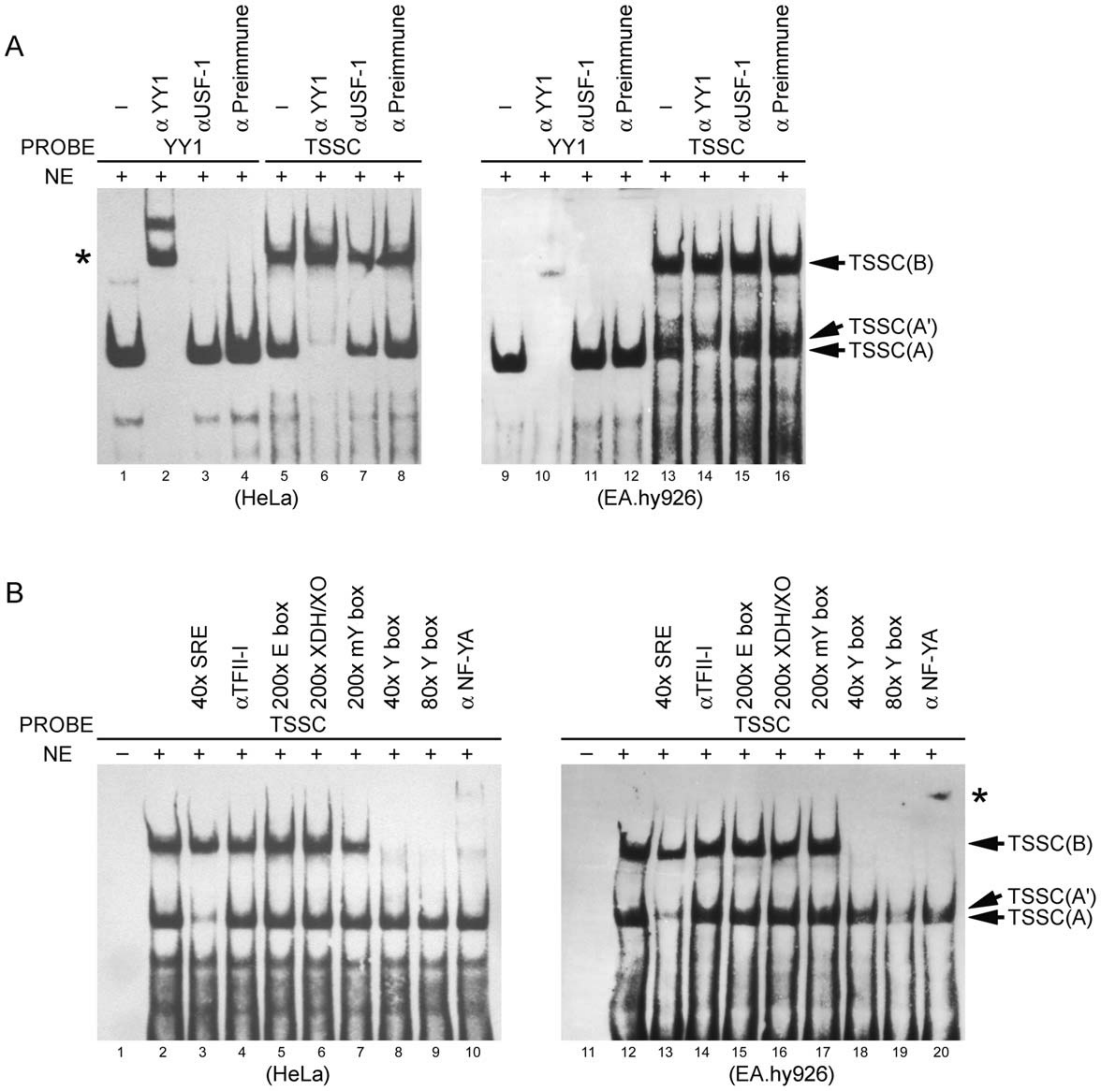
YY1 and NF-Y bind to the TSSC element of the exon 1a promoter. (**A**) and (**B**) HeLa or EA.hy926 cell nuclear extract (NE) was incubated with a labeled probe containing the TSSC of the exon 1a promoter and the reaction mixture was electrophoresed on a 4.5% non-denaturing gel to detect the specifically retarded migrating band. The indicated unlabeled probes or antibodies were added prior to labeled probes for competition or super-shift analysis. The canonical YY1 oligonucleotide derived from the SVCT2 exon 1b promoter served as a positive control. A super-shifted complex is indicated by an *. Note: TSSC(A′), unidentified binding complex.

**Figure 2:**
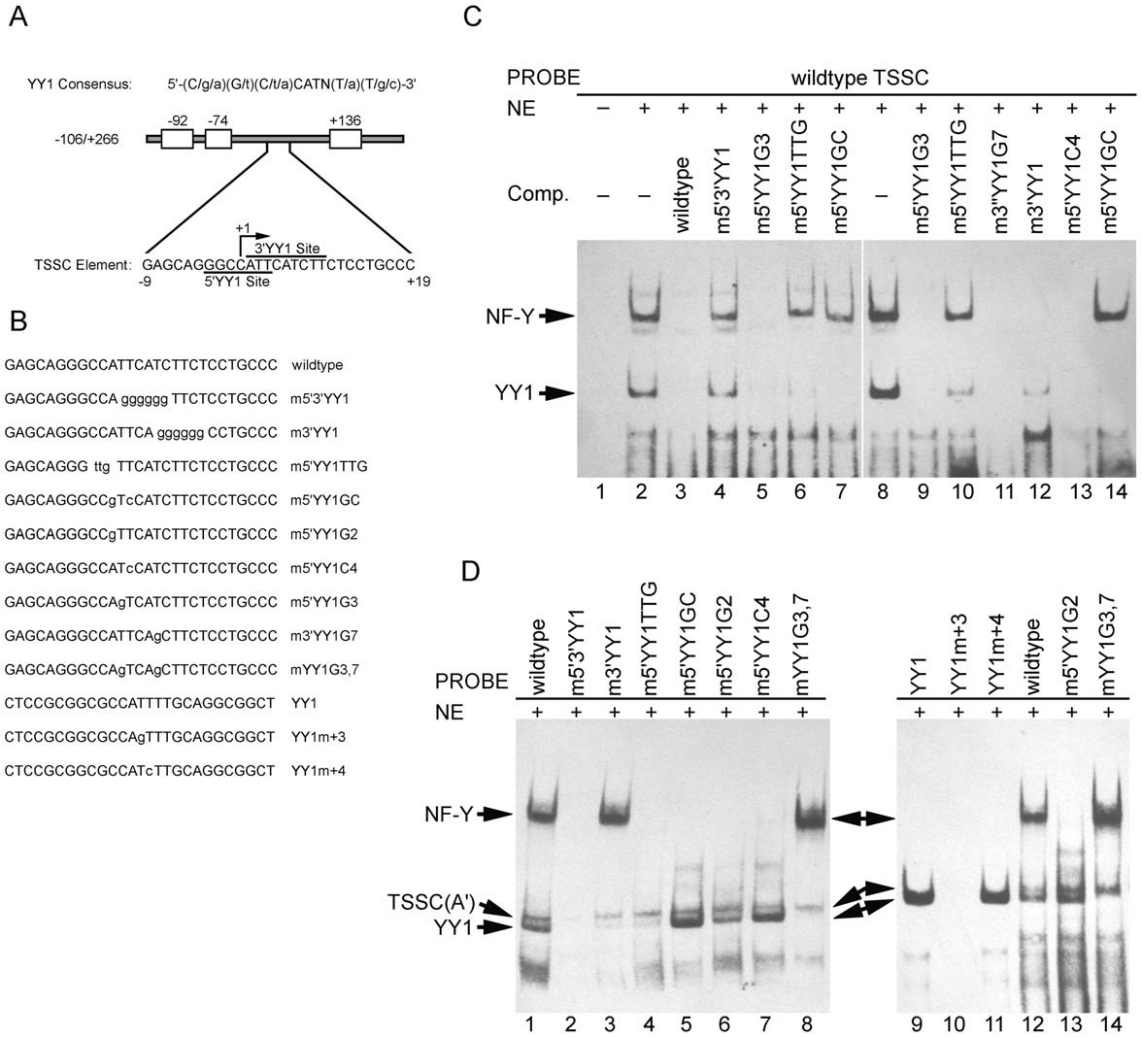
Characterization of the TSSC element of the exon 1a promoter. (**A**) The consensus sequence for YY1 binding is shown, where the upper case letters represent the preferred bases. The YY1 binding site contains a conserved core 5′-CAT-3′, which is essential for efficient binding, and is flanked on either side by variable regions. Two potential binding sites for YY1 were identified within the TSSC element and each putative binding site for YY1 is marked. (**C**) Competition gel shift assays were performed with HeLa nuclear extract to define the binding specificity of YY1 and NF-Y to the TSSC element. Each competitor used was added in a 200-fold molar excess. The applied competitors with mutations in the YY1 consensus site are described in (**B**) and **Table 1**. (**D**) Ability of the TSSC mutant oligonucleotides to form YY1 or NF-Y complexes. Wild type TSSC and 5′ and/or 3′YY1 mutant oligonucleotides were labeled and used in EMSAs with HeLa nuclear extract. Note: TSSC(A′), unidentified binding complex.

To evaluate the role of the TSSC downstream of functional NF-Y/USF elements in the regulation of the exon 1a promoter, we used gel-shift assays to detect additional transcription factors binding to this DNA region. We observed two major protein-DNA complexes, designated TSSC(A), and TSSC(B), binding to this element (Fig. 1A and B). Inspection of the TSSC sequence identified two potential binding sites for the multifunctional transcription factor YY1 [13]. YY1 has been shown to bind to sequences overlapping transcription start sites, where it functions to activate transcription [14], [15], [16]. As shown in Fig. 2A, the TSSC contains two copies of the core sequence 5′-CAT-3′ that individually are necessary for YY1 binding [16], [17]. Based on the sequence similarity between the TSSC and YY1 binding sites, we explored the possibility that YY1 is a component of these complexes. As shown in Fig. 1A, the TSSC(A) complex was shifted to a position co-migrating with the TSSC(B) complex upon addition of YY1 antibody, but unaffected by addition of antibody directed against USF1 or by the preimmune serum. The upper complex, TSSC(B), was not affected. The canonical YY1 oligonucleotide derived from the SVCT2 exon 1b promoter served as a positive control. These data confirm that TSSC(A) complex contains YY1 protein. Similar results were observed in both human derived cell lines, HeLa and EA.hy926.

The TSSC of the exon 1a promoter contains an almost perfect match (7 out of 8 residues) to the TFII-I motif of 5′-YAYTCYYY-3′ (where Y is a pyrimidine residue) (Fig. 2A) [18]. However, excess of a known TFII-I binding sequence from the serum response element (SRE) did not disturb the TSSC(B) complex (Fig. 1B). TFII-I antibody also failed to affect the TSSC(B) band, nor did it produce a super-shifted band. This suggests that TFII-I does not bind to the TSSC(B).

Rigby and his colleagues have previously shown that NF-Y is able to bind both the HoxTF/YY1 site (TGGCCATT) [19] and the b4Cwt site (TCGCCATT) [20] in the Hoxb4 promoter. As shown in Fig. 1B, a 29-bp competitor oligonucleotide containing a canonical CCAAT binding site (Y box) derived from the SVCT2 exon 1a promoter [12] competed efficiently for TSSC(B) binding without affecting YY1 binding, whereas the nonspecific competitor (XDH/XO), exon 1a promoter-derived E box [12] and the mutated Y box failed to compete. Finally, the retarded band observed with the TSSC was completely super-shifted with the anti-NF-YA antibody, verifying that the NF-Y trimer has TSSC(B) binding activity (Fig. 1B). Again, comparable results were observed for both HeLa and EA.hy926 cells.

### The 3′YY1 binding site exclusively binds YY1, whereas the 5′YY1 binding site binds both NF-Y and YY1

We have demonstrated that the TSSC(A) and TSSC(B) DNA-protein complexes contain YY1 and NF-Y, respectively. The TSSC element contains two overlapping sequences with homology to the YY1 consensus binding site (Fig. 2A). Both the 5′ and 3′YY1 sites are identical to the invariant core sequence (5′-CAT-3′) of the YY1 consensus motif [17]. To determine the site binding specificity of YY1/NF-Y within the TSSC element, oligonucleotides containing specific mutations of the core consensus sequence in either the 5′YY1 site (m5′YY1GC, m5′YY1G2, m5′YY1G3, m5′YY1TTG, and m5′YY1C4), the 3′YY1 site (m3′YY1 and m3′YY1G7), or both sites (m5′3′YY1 and mYY1G3,7) of the TSSC element of the exon 1a promoter were engineered (Fig. 2B and Table 1).

**Table 1:** Probes used in the current study.

Name	Sequence
YY1	5-CTCCGCGGCGCCATTTTGCAGGCGGCT-3
TSSC	5- GAGCAGGGCCATTCATCTTCTCCTGCCC-3
XDH/XO	5-CCGGGAGGCGTATCTTTCAAGTTGCAGGGCAGT-3
SRE	5- AATTCTCCTTTACACAGGATGTCCATATTAGGACATCTC-3
E box	5-TCCACTTTCACCCACGTGAGCAGGCATCAT-3
Y box	5-AGCAGGCATCATCCAATCCACTGTGGGTC-3
mY box	5-AGCAGGCATCATCCAGGCCACTGTGGGTC-3
YY1m+3	5-CTCCGCGGCGCCAGTTTGCAGGCGGCT-3
YY1m+4	5-CTCCGCGGCGCCATCTTGCAGGCGGCT-3
m5′3′YY1	5-GAGCAGGGCCAGGGGGGTTCTCCTGCCC-3
m3′YY1	5-GAGCAGGGCCATTCAGGGGGGCCTGCCC-3
m5′YY1TTG	5-GAGCAGGGTTGTTCATCTTCTCCTGCCC-3
m5′YY1GC	5-GAGCAGGGCCGTCCATCTTCTCCTGCCC-3
m5′YY1G2	5-GAGCAGGGCCGTTCATCTTCTCCTGCCC-3
m5′YY1C4	5-GAGCAGGGCCATCCATCTTCTCCTGCCC-3
m5′YY1G3	5-GAGCAGGGCCAGTCATCTTCTCCTGCCC-3
m3′YY1G7	5-GAGCAGGGCCATTCAGCTTCTCCTGCCC-3
mYY1G3,7	5-GAGCAGGGCCAGTCAGCTTCTCCTGCCC-3

We tested the capacity of these oligonucleotides to compete for the binding of nuclear YY1 and NF-Y to the wild-type TSSC sequence. As shown in Fig. 2C, addition of excessive wild-type sequence completely abolished both complexes (lane 3), whereas the double mutant m5′3′YY1 was unable to disrupt either YY1 or NF-Y (lane 4). The competitors m5′YY1TTG and m5′YY1GC also failed to compete effectively for NF-Y binding (lanes 6, 7, 10 and 14) suggesting that NF-Y specifically recognizes the 5′YY1 binding site within the TSSC element. However, the m5′YY1TTG mutant partially competed for YY1 binding, indicating that the TTG mutation not only eliminated NF-Y binding, but also reduced YY1 activity. Javahery et al has demonstrated that a change of the YY1 core consensus from CCAT to ttgT completely eliminates YY1 binding and appears not to affect the 3′YY1 binding site [16]. This indicates that part of YY1 binding activity appears to derive from the 5′YY1 site. Consistent with this observation, the mutation of the 3′YY1 site (m3′YY1) disrupted most of YY1 binding and abolished NF-Y binding (lane 12). The m5′YY1GC and m5′YY1C4 mutant sequences completely abolished YY1 binding, probably because of the production of a high affinity binding site (CCATCTT) for YY1 (lanes 7, 13 and 14). Moreover, in contrast to the positive control (Fig. 2D, lane 10), the competitors bearing a critical nucleotide mutation for YY1 binding sites (m5′YY1G3 and m3′YY1G7) (Fig. 2D, lane 10) [11], [16], [21] still completely eliminated YY1 and NF-Y complexes (Fig. 2C, lanes 5, 9 and 11). This further suggests that either the 5′ or the 3′YY1 binding site is capable of binding YY1. These results provide evidence that NF-Y specifically recognizes the 5′YY1 binding site within the TSSC element and that YY1 is capable of binding either the 5′YY1 or the 3′YY1 site.

To directly test the ability of nuclear extracts containing YY1/NF-Y to bind the 5′/3′YY1 sites, EMSA was performed with the labeled mutant oligonucleotides described above. The results of these experiments are shown in Fig. 2D. Mutation of both YY1 sites eliminated the TSSC binding (lane 2). The oligonucleotide with a mutated 3′YY1 site (m3′YY1) eliminated most of YY1 binding activity, but did not affect NF-Y binding activity (lane 3). Several mutations in the 5′YY1 site were also tested. A single point mutation of the 5′YY1 site at +2 from A to G (m5′YY1G2) abolished NF-Y binding activity without affecting the binding of YY1 (lanes 6 and 13), and another point mutation at +4 from T to C (m5′YY1C4) severely impaired NF-Y binding activity and improved YY1 binding (lane 7), indicating that the 3′YY1 site contributes to YY1 binding since +4 mutation does not impair 5′ YY1 binding (lane 11) [11]. The combined mutation (m5′YY1GC) completely eliminated NF-Y binding activity and produced a high affinity binding site for YY1 (lane 5). The mutant m5′YY1TTG still retained weak binding for YY1 (lane 4). In addition, the mutant bearing a critical nucleotide mutation in each of YY1 binding sites (mYY1G3,7) completely abolished YY1 binding and increased NF-Y binding (lanes 8 and 14). Taken together, the observations from Fig. 2 strongly support the notion that NF-Y specifically recognizes the 5′YY1 binding site and YY1 is capable of recognizing either the 5′YY1 or the 3′YY1 binding site.

### YY1-mediated transcriptional activation of the exon 1a promoter requires the integrity of two adjacent YY1 sites to maintain the optimal promoter activity

**Figure 3:**
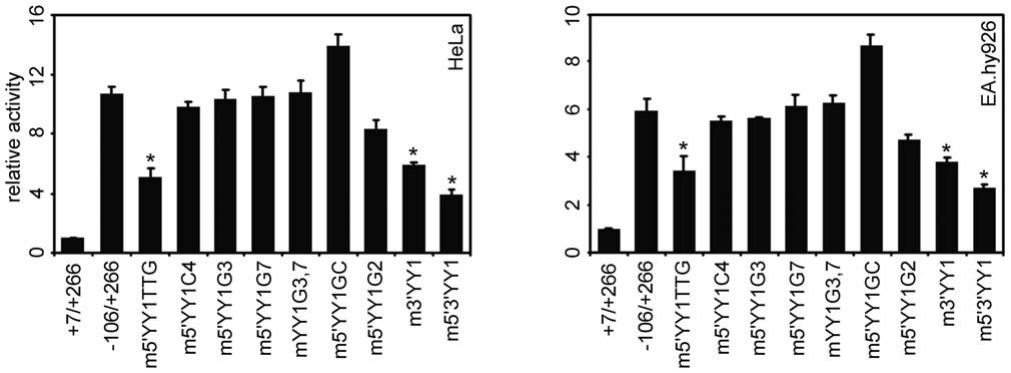
Effect of 5′/3′YY1 mutations on the exon 1a promoter transcriptional activity. HeLa and EA.hy926 cells were transfected with 500 ng of TSSC mutants described in Fig. 2B and Table 1. 5 ng of pRL-CMV was added as an internal control for each transfection, and relative luciferase activity is shown as the means based on the activity of +7/+266. Data represent mean ± S.E.M. (n = 4). *P<0.05 versus −106/+266.

To further evaluate the role of the two YY1 sites and YY1/NF-Y transcription factors in the regulation of the exon 1a promoter activity, we tested the capacity of the exon 1a reporter constructs carrying the various mutations in both YY1 binding sites (as depicted in Fig. 2B and Table 1) to affect the exon 1a promoter activity. As shown in Fig. 3, the mutants m5′YY1TTG and m5′3′YY1 both decreased the promoter activity by about 50–70%, consistent with the patterns of impaired YY1/NF-Y binding. Improved NF-Y binding maintained exon 1a promoter activity in the partial or complete absence of YY1 binding (m5′YY1G3, m3′YY1G7 and mYY1G3,7), and vice versa (m5′YY1GC and m5′YY1C4). In contrast, m3′YY1, which impairs the binding of YY1, but does not significantly affect NF-Y binding, significantly decreased exon 1a promoter activity. The mutant (m5′YY1G2) that impairs NF-Y binding without affecting YY1 binding slightly decreased promoter activity. These data suggest that both YY1 sites within the TSSC element are required to maintain the maximal promoter activity and that positive regulation is mediated through the binding of YY1 and NF-Y.

**Figure 4:**
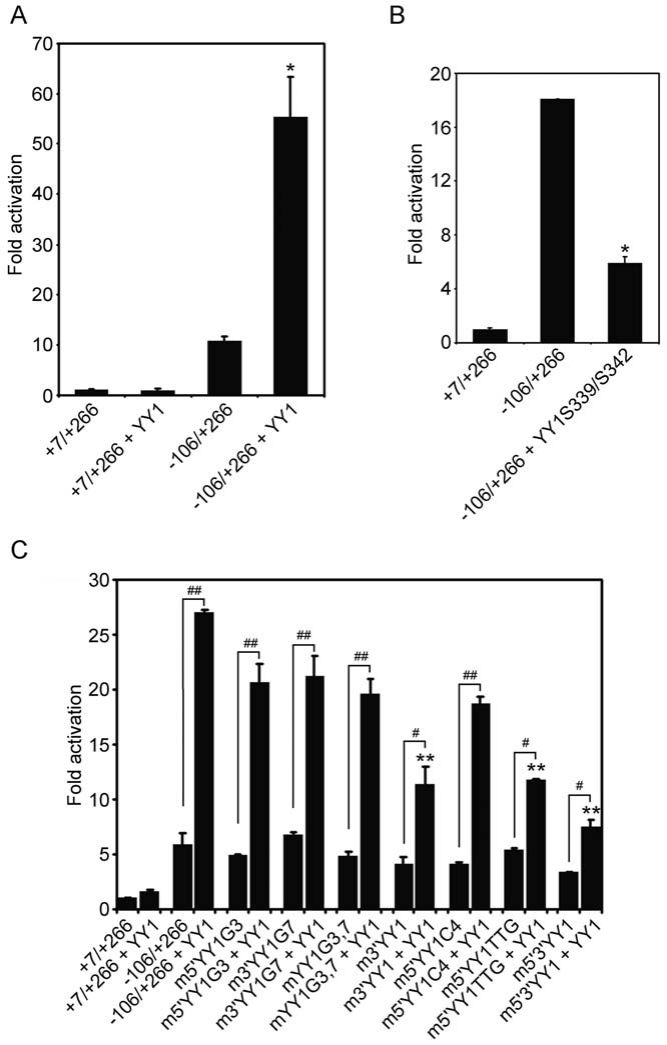
Trans-activation of the exon 1a promoter by YY1. (**A**) and (**B**) HeLa cells were co-transfected with 100 ng of the exon 1a reporter plasmid (−106/+266-Luc) along with 400 ng of the plasmid containing YY1 expression plasmid, or 200 ng of promoter constructs with 300 ng of the dominant-negative mutant YY1S339/S342 as indicated. (**C**) Effect of mutation of YY1 binding sites on YY1 induction of luciferase activity under the control of the exon 1a promoter. 100 ng of the exon 1a mutants containing mutations as described in Fig. 2B and Table 1 were transfected into HeLa cells with or without 400 ng of the YY1 construct. Luciferase activity was measured 24 h after transfection. Means ± S.E.M. are shown (n = 4). *P<0.01 versus −106/+266; **P<0.01 versus −106/+266 + YY1; #*P*<0.05; ##*P*<0.01.

To directly confirm that YY1 exerts a positive effect on the exon 1a promoter, we tested whether transfection of YY1 could trans-activate the exon 1a promoter. As shown in Fig. 4A, a significant response was observed with maximal stimulation of YY1 expression resulting in a 5–6-fold activation. As an additional control, the low level of expression observed with the empty reporter vector was not altered by YY1. To further demonstrate the involvement of YY1 in the exon 1a promoter activation, a dominant-negative YY1 mutant (YY1S339/S342) was employed to interfere with binding of the endogenous YY1 to the exon 1a promoter. This mutant lacks the ability to bind specific YY1-target sequences, but retains the capability of wild type protein for protein–protein interactions [22]. As shown in Fig. 4B, basal transcription was substantially inhibited by ectopically expressed YY1S339/S342. These data further confirm that YY1 plays a positive regulatory role in the transcription of the exon 1a promoter.

To elucidate the role of NF-Y in YY1-mediated trans-activation of the exon 1a promoter, reporter gene expression was evaluated in transient transfection assays using YY1 construct and exon 1a promoter mutants with mutations as described in Fig. 2B and Table 1. As observed in Fig. 4A, Fig. 4C shows that the wild type exon 1a promoter (−106/+266) was up-regulated 4.6-fold in response to the exogenous expression of YY1. When the YY1 binding sites were mutated and YY1/NF-Y binding was completely eliminated (m5′3′YY1), YY1 still stimulated exon 1a promoter activity by two-fold, although YY1 activation was attenuated nearly 4-fold compared with the wild type control construct (Fig. 4C). When YY1 binding was severely impaired with increased NF-Y binding (m5′YY1G3, m3′YY1G7 and mYY1G3,7), or NF-Y binding was nearly eliminated with increased YY1 binding (m5′YY1C4), YY1 activation of the exon 1a promoter was slightly decreased. Promoter activation occurred even when YY1 binding was completely eliminated (mYY1G3,7). These results suggest that TSSC-bound NF-Y may contribute to YY1-mediated induction of the exon 1a promoter. The m5′YY1TTG or m3′YY1 mutants resulted in only two-fold YY1 activation (less than 40% of wild type). These results suggest that YY1-mediated trans-activation of the exon 1a promoter requires the integrity of the NF-Y binding site as well as the YY1 binding sites within TSSC element. Our finding that YY1 was still able to activate the exon 1a promoter despite of the absence of the YY1 binding site raises the possibility that YY1 might interact with other factors directly or indirectly bound to the exon 1a promoter in the absence of YY1 binding site. Owing to the key role of the upstream Y box and its bound NF-Y, we are unable to definitively assign any function to TSSC-bound NF-Y, although our results suggest a positive regulation on the exon 1a promoter activity.

### YY1 synergistically activate the exon 1a promoter along with NF-Y and USF

**Figure 5:**
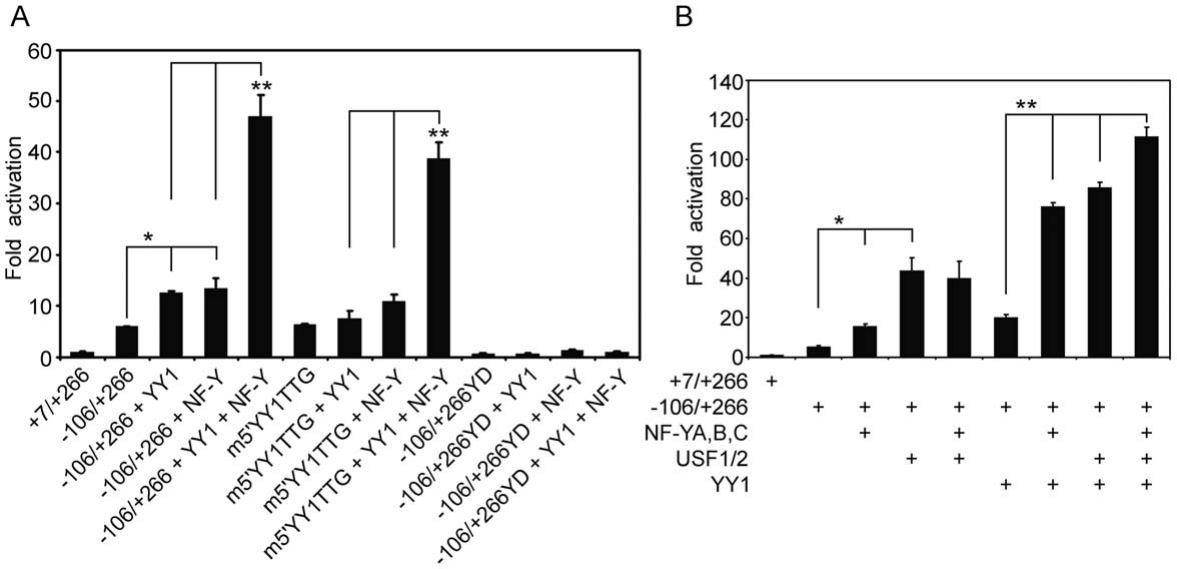
YY1, NF-Y and USF synergism on the exon 1a promoter. (**A**) NF-Y synergistically potentiates YY1 induction on the exon 1a promoter. HeLa cells were transiently transfected with 100 ng of −106/+266, m5′YY1TTG, or −106/+266YD (the central CCAAT sequence changed to CCT) reporter constructs in the presence of 200 ng of NF-Y (equimolar mixtures), or/and YY1 constructs. (**B**) Cells were transiently transfected with 100 ng of −106/+266 reporter construct in the presence of 300 ng of the expression vectors for NF-Y, USF1/2, or/and YY1. Relative luciferase activity is shown as the means based on the activity of +7/+266. Means ± S.E.M. are shown (n = 4). *P<0.05; **P<0.01.

In our previous study, we showed that NF-Y bound to the Y box interacts with USF1/2 bound to the E box on the upstream promoter, and that the formation of the NF-Y/USF complex is absolutely required for the full activity of the exon 1a promoter [12]. To further investigate the functional relationship between YY1, NF-Y and USF in regulating the exon 1a promoter activity, YY1, NF-Y and USF constructs were co-transfected with the exon 1a promoter into HeLa cells. As shown in Fig. 5A, transfection with either YY1 or NF-Y alone increased promoter activity by two-fold. On the other hand, co-transfection of YY1 with NF-Y synergistically increased promoter activity to 8-fold. The 5′YY1 site mutation (m5′YY1TTG), which impairs YY1 binding and completely abolishes NF-Y binding, still produced a synergistic response to the combination of NF-Y and YY1. This suggests that the Y box located in the −74/−70 region and its bound NF-Y play a critical role in the YY1-induced response. In contrast, the mutation of this Y box (−106/+266YD) [12] abrogated any response to YY1 and/or NF-Y (Fig. 5A).

As with NF-Y, co-transfection of YY1 with USF1/2 also synergistically increased the promoter activity to 16-fold over that of the intact promoter alone (Fig. 5B). This was a level that was twice that of USF1/2 alone. However, NF-Y failed to synergistically enhance USF1/2-mediated activation, although NF-Y and USF bind to the exon 1a promoter in a cooperativity-dependent manner [12]. On the other hand, the combination of YY1, NF-Y and USF1/2 dramatically enhanced the promoter activity to 21-fold compared to the −106/+266 construct alone. These observations show that NF-Y, USF and YY1 synergistically activate the exon 1a promoter activity compared to each factor alone.

### p300 synergistically regulates the exon 1a promoter activity via the interaction with other transcription factors

**Figure 6:**
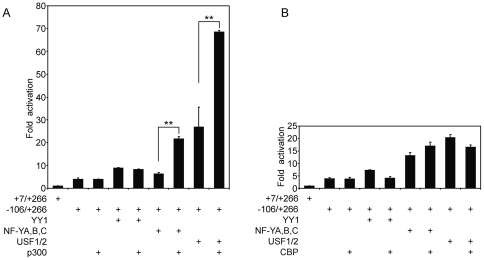
Non-DNA-binding transcriptional co-activator p300 is involved in the transcriptional regulation of the exon 1a promoter and synergistically enhances promoter activity in conjunction with other transcription factors. HeLa cells were transiently transfected with 100 ng of −106/+266 reporter constructs in the presence of 600 ng of the expression vectors for p300 (**A**) or CBP (**B**) with 300 ng of YY1, NF-Y (equimolar mixtures) or USF1/2 (equimolar mixtures). The data are shown as means ± S.E.M. (n = 4), with the response level of +7/+266 reporter construct in the absence of NF-Y, YY1 and p300/CBP set arbitrarily as 1. **P<0.01.

The preceding results show that YY1-mediated induction of the exon 1a promoter is dependent on the integrity of NF-Y/USF binding sites upstream of the TSSC element and that YY1 and NF-Y/USF synergistically activate the exon 1a promoter. Although this could reflect a protein-protein interaction between YY1 and USF/NF-Y, it does not prove one, since it is possible that one or more co-activators mediate this effect. It has been shown that the p300 co-activator can interact with YY1/NF-Y/USF and facilitate transcriptional activation [23], [24], [25]. As shown in Fig. 6A, p300 had no effect on the basal promoter activity when expressed alone and failed to enhance YY1-mediated promoter activity. On the other hand, the increase in promoter activity with either NF-Y or USF alone was further elevated when p300 was also expressed with each of them (Fig. 6A). That the effect of p300 was specific is evident from the results shown in Fig. 6B, in which, its closely related family member CBP had little or no effect on the exon 1a promoter activity.

### Binding of YY1, NF-Y and p300 to the proximal exon 1a promoter in cells

**Figure 7:**
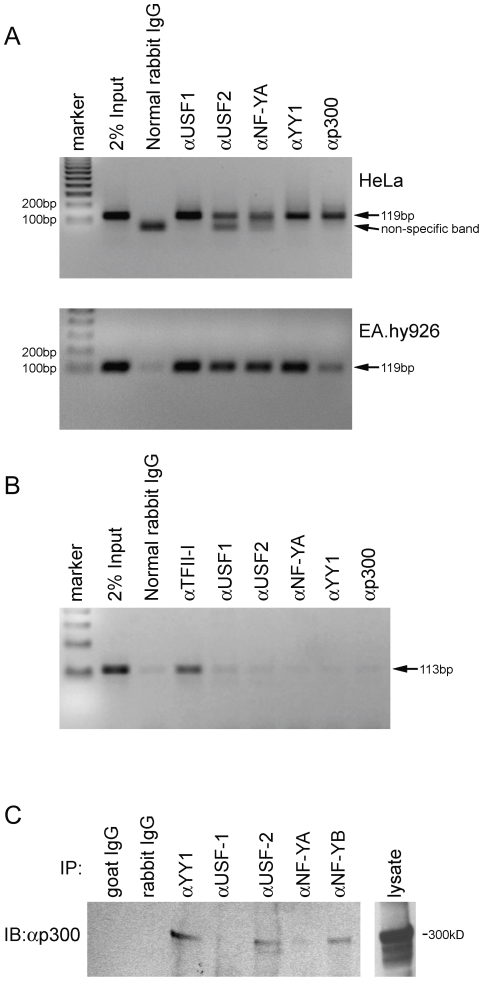
Analysis of transcription factor binding to the exon 1a promoter. (**A**) ChIP assays were performed using HeLa and EA.hy926 cells. Antibodies for YY1, NF-Y, USF1/2 and p300 or normal rabbit IgG were used. Immunoprecipitated DNA fragments and 2% of total sample DNA were amplified by PCR using primers specific for the human exon 1a promoter (−106 to +13). PCR products were separated on a 2% agarose gel and stained by ethidium bromide. (**B**) Amplification of an unrelated region showed undetectable signal from HeLa cell-derived genomic DNA. (**C**) Co-immunoprecipitation assay showing the interactions with p300. HeLa cells were lysed and immunoprecipitated using YY1, USF1, USF2, NF-YA or NF-YB antibodies followed by immunoblotting with anti-p300 antibody.

Chromatin immunoprecipitation (ChIP) assays were used to directly assess the presence or absence of YY1, NF-Y and p300 bound to the proximal promoter of the endogenous exon 1a gene. As shown in Fig. 7A, a 119-bp fragment spanning the exon 1a proximal promoter was detected by PCR in 2% Input. When DNA from HeLa or EA.hy926 cells was immunoprecipitated with antibodies to USF1/2, NF-YA, YY1 and p300, the 119-bp region was detected. As a control for non-specific protein-DNA interactions, we also amplified a genomic fragment containing a TFII-I site where no sites for other transcription factors are detectable by sequence analysis. The resulting 113 bp fragment from HeLa cells was immunoprecipitated by TFII-I antibody, but not significantly by antibodies to USF1/2, NF-YA, YY1 or p300 (Fig. 7B). Furthermore, immunoblotting of p300 after immunoprecipitation with antibodies to YY1, USF2 and NF-YB showed that the immunoprecipitates contained p300, but neither USF1 nor NF-YA did (Fig. 7C), suggesting that p300 is capable of mediating the protein-protein interaction among YY1, NF-Y and USF1/2.

**Figure 8:**
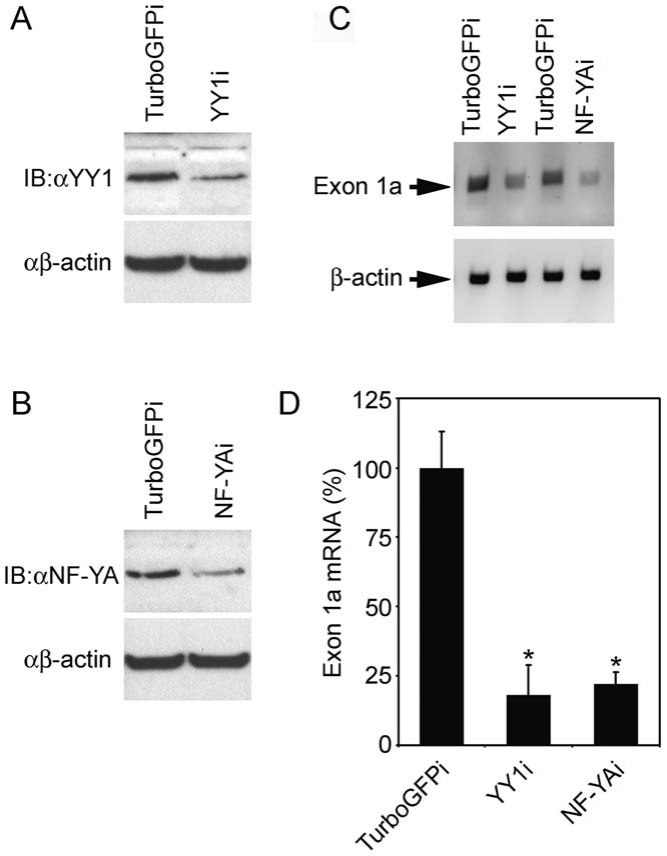
Effect of NF-Y and YY1 silencing on the SVCT2 exon 1a expression. (**A**) and (**B**) YY1, NF-YA and β-actin protein level of EA.hy926 cells transduced with pLKO.1-NF-YAi, pLKO.1-YY1i or pLKO.1-TurboGFPi lentivirus was immunoblotted with specific antibodies. (**C**) The SVCT2 exon 1a and β-actin mRNA level of EA.hy926 cells, transduced as above, was determined by RT-PCR. Data are representative of three independent experiments. (**D**) The data is a densitometric analysis of 3 independent experiments as means ± S.E.M. (n = 3). *P<0.05, as compared with control cells.

To further demonstrate the relationship between these transcription factors and the SVCT2 exon 1a transcript expression, EA.hy926 cells were transduced with the lentivirus RNAi pLKO.1-YY1 or NF-YA vectors to silence YY1 or NF-YA. Four days after transduction, total protein and mRNA were extracted from EA.hy926 cells and used to determine YY1/NF-YA protein levels and SVCT2 exon 1a mRNA level. As shown in Figure 8A and B, YY1 or NF-YA silencing significantly decreased YY1 or NF-YA protein levels. Parallel to YY1 or NF-YA silencing, the exon 1a transcript level was markedly decreased as compared to those in control cells (Figure 8C and D).

Together, these results provide evidence for the direct involvement of these transcription factors in the regulation of the SVCT2 exon 1a expression.

## Discussion

Although the critical *cis*-acting DNA elements (E box and Y box) required for SVCT2 exon 1a promoter activity have been determined [12], important core promoter elements have not been described. Clearly, elucidation of transcriptional regulatory mechanisms relies on thorough characterization of promoter sequence. To understand the potent transactivation potential of E box-bound USF and Y box-bound NF-Y, we have further characterized the exon 1a promoter to identify possible targets for regulation within the basal transcriptional machinery. Here we identified a core region, the TSSC element, which encompasses the transcription start site and is required for promoter activity. To identify factors that might regulate the exon 1a expression through the TSSC element, we inspected the TSSC sequence for homology to well-characterized initiator-binding proteins. The TSSC contains two copies of the core sequence 5′-CAT-3′, which is predicted to be recognized by the YY1 transcription factor [17], [26], [27]. Using immunological techniques, we confirmed that YY1 is a component of one of the TSSC complexes formed on the exon 1a promoter. YY1 is a multifunctional transcription factor that can exert either positive or negative control on a large number of cellular and viral genes by binding to sites overlapping the transcription start site. It is ubiquitously expressed and is highly conserved between mouse and human [13]. YY1 regulates the expression of a variety of cellular and viral genes such as the adeno-associated virus P5 promoter (AAV P5) and the cytochrome oxidase Vβ subunit promoter (COX Vβ) [14], [15], [28].

We found that the exon 1a promoter activity relies on maintenance of the two intact YY1 binding sites, since specific mutations within the YY1 core consensus sequence that prohibit YY1 binding activity also impaired the transcriptional response of the exon 1a promoter. The YY1 protein positively regulates the exon 1a promoter, since overexpression of YY1 enhanced transcriptional activity. We also observed that YY1 is capable of binding to either of 5′/3′YY1 binding sites in a functional manner. However, the two closely adjacent YY1 binding sites seem highly unlikely to be recognized by two YY1 molecules simultaneously because of the requirement for flanking residues. The latter conclusion derives from methylation analysis of the canonical YY1 sequence derived from SVCT2 exon 1b promoter (data not shown) and the first intron of Peg3 [29].

We also identified the 5′YY1 binding site as an NF-Y/YY1 motif (GCCATT) within the TSSC element of the exon 1a that includes the two adjacent potential YY1 binding sites (_5-GCCATT_ ^CATCTT^ ^-3^). The NF-Y/YY1 motif can act as a potential binding site for NF-Y. Previously, Gilthorpe et al [20] also described a similar NF-Y/YY1 motif (TCGCCATT) within Hoxb4 intron C1 region that bound NF-Y and YY1 in mouse embryo and neuro 2A nuclear extracts. NF-Y binding to the NF-Y/YY1 motif is important for region C enhancer activity of Hoxb4 in both mesodermal and neural domains and positive regulation is largely mediated through the binding of NF-Y [20]. However, it seems unlikely that both NF-Y and YY1 can bind simultaneously because of the requirement for common major groove interactions [17], [20], [30]. It would appear, therefore, that the NF-Y/YY1 site is a specialized motif that is able to bind either factor in a mutually exclusive fashion.

NF-Y is known to stabilize the binding of other proteins to regulatory elements close to CCAAT boxes and to interact directly with other transcription factors. These properties of NF-Y could be important for the recruitment of additional proteins to the promoter in order to establish complexes that are capable of activating transcription. NF-Y can also interact with proteins of the general transcriptional machinery [31]. In our recent work, the NF-Y transcription factor was found to increase expression of SVCT2 exon 1a through its binding to the CCAAT Y box motif on the exon 1a promoter in cooperation with USF1/2 binding to the upstream E box [12]. Thus, we speculate that, in cooperation with upstream DNA-USF/NF-Y complex, NF-Y binding to the NF-Y/YY1 motif might act to stabilize and enhance the activating effects of other cell-type specific transcriptional regulators, maintain this state by the recruitment of chromatin modifying enzymes, and indirectly stabilize and enhance YY1 binding. This is also supported by the fact that YY1 still strongly stimulated the exon 1a promoter activity in the absence of the YY1 binding site. Although our current analysis supports the possibility that regulation of the exon 1a expression might be influenced by this atypical CCAAT box-bound NF-Y and YY1 simultaneously, given the overlapping nature, we prefer the following hypothesis. Since NF-Y and YY1 can mediate different transcriptional effects by reorganizing the local chromatin environment, the relative levels of NF-Y and YY1 binding could represent a balancing mechanism for exon 1a activity through the specialized TSSC and this mechanism might also be involved in the spatially specific expression of the exon 1a [20].

However, there is lack of evidence demonstrating that YY1 can directly interact with NF-Y subunits. Here the transcriptional co-activator p300, but not its closely related family member CBP, synergistically increased the exon 1a promoter activity via the interaction with other transcription factors and thus could serve as a “bridge” for transcription factor interaction. The p300 protein is a non-DNA-binding transcriptional co-activator that interacts with transcriptional factors and is implicated in transcriptional responses to various extracellular and intracellular signals. It functions by chromatin remodeling and is involved in most cellular programs, including growth, terminal differentiation, and p53-mediated apoptosis. Further, p300 is also involved in the activation of a large number, if not all, the polymerase II-transcribed genes. Activity of p300 has been studied in a number of systems, with particular focus on its acetyltransferase enzymatic activity. Three different functions are attributed to p300. First, the protein serves as a platform, a bridge, through which the direct interactions with multiple DNA binding activators are supported; recruitment of p300 stabilizes and increases the otherwise weak binding of these factors or even makes them possible [32]. Interactions of p300 with many transcription factors have been mapped in one and sometimes multiple sub-domains of the co-activator (e.g., NF-Y binding) [24]. Second, once on a promoter, p300 protein modifies the chromatin structures nearby the sites by virtue of its histone acetylation activity, rendering nucleosomes more “accessible” to the general transcription apparatus. Third, the same histone acetyltransferase activity increases the affinity of the DNA binding factor for the targeted sequence and modulates transcriptional activity. In keeping with this, p300 can acetylate NF-YB and this modification increases NF-Y-p300 interactions [33]. Considering the fact that p300 is capable of interacting with YY1, NF-Y and USF, respectively, the “bridging” mechanism might be predominant for the SVCT2 exon 1a promoter. It is also possible that the acetylation modification of NF-YB by p300 favors the interactions of NF-Y-p300-NF-Y and increases the binding of NF-Y to the canonical Y box and atypical NF-Y/YY1 motif [34], [35], [36].

Our findings revealed that all of these transcription factors are bound to their cognate sites on the endogenous SVCT2 exon 1a promoter in both exon 1a-expressing cells (EA.hy926) and non-expressing cells (HeLa) and the exon 1a promoter also exhibited similar transcriptional activity in both types of cell lines [12]. In this regard, previous studies implied that the total transcription lack requires a local repression mechanism to prevent activation by these ubiquitous transcription factors and this local inhibition appears to depend on the promoter activity [37], [38], [39]. The promoter 5′ region protection against remethylation is probably related to the transcriptional activity of the promoter and the promoter-bound transcription factors. Transcription factors may either directly prevent access of the DNA methyltransfrase or indirectly induce histone modifications that exclude these methylation enzymes against methylation [40], [41], [42]. Consistent with this, our current study confirmed that p300 is involved in the transcriptional regulation of the exon 1a. This co-activator is capable of interacting with NF-Y/USF functional complex and synergistically increased the exon 1a promoter activity. The p300 co-activator can function by relaxing the chromatin structure at the gene promoter through their intrinsic histone acetyltransferase (HAT) activity. Thus, the exon 1a 5′ region could be protected against remethylation in non-expressing cells due to the presence of these transcription factors.

**Figure 9:**
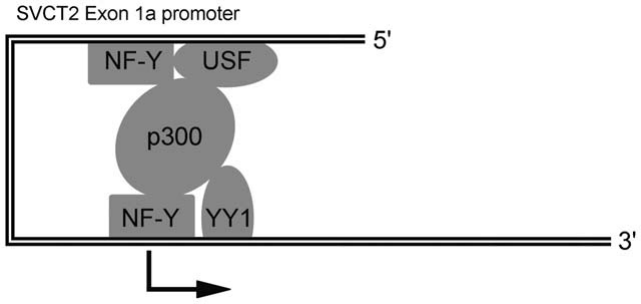
Proposed model for the SVCT2 exon 1a promoter trans-activation. p300 of the transcriptional complex could act as a bridging factor among YY1/NF-Y/USF.

These results suggest that these transcription factors might interact as a complex on the SVCT2 exon 1a promoter via a “bridging” mechanism. Based on it, we propose a simplified possible model for SVCT2 exon 1a promoter activation by YY1, NF-Y and USF. Fig. 9 shows how these transcription factors could converge to form the transcriptional complex required for the exon 1a gene transcription. First, Y box-bound NF-Y and E box-bound USF form an activating complex on the upstream of the exon 1a promoter. Second, the NF-Y/USF protein complex on the promoter recruits the p300 co-activator. Third, p300 provides a crucial platform to bind YY1 and NF-Y present in the TSSC element and also bends the DNA to facilitate the assembly of the transcriptional complex. Finally, the co-activator p300 may also interact with other transcription factors or modify histones. The latter probably represents a critical event for the achievement of an open chromatin state that could also favor the formation of the transcriptional complex on the exon 1a promoter.

The experiments reported in this study also demonstrate that these transcription factors control the cellular expression of SVCT2 exon 1a and presumably expression of the SVCT2 itself. Lentivirus-mediated knockdown of NF-YA- or YY1 significantly impaired exon 1a mRNA expression. Such transcriptional regulation of SVCT2 expression could well contribute to changes in SVCT2 protein expression and thus vitamin C uptake. For example, brain capillary endothelial cells in primary culture do not express the SVCT2, yet develop begin to express it during the process of cell culturing and after transient murine stroke [43], [44]. In both cases, this may be due to increased oxidative stress. Further, phorbol ester-induced differentiation of THP-1 monocytes caused a marked increase in both expression and function of the SVCT2, leading to increases in intracellular ascorbate [6]. Similar increases in ascorbate facilitated THP-1 monocyte adhesion and morphologic differentiation induced by low concentrations of PMA, and decreased the expression of the monocyte-macrophage surface antigen CD14 [6]. Since such changes in SVCT2 expression may well be due to changes in SVCT2 transcription, study of the transcriptional regulatory mechanisms of SVCT2 could provide new insight on how SVCT2 expression might be regulated upon the different stimuli and environments and contribute to the elucidation of SVCT2's role against oxidative stress [6], [11], [12].

## Materials and Methods

### Reagents

The antibodies against USF1 (C-20), USF2 (C-20), YY1 (H-414), TFII-I (H-58), p300 (NM11), NF-YA (C-18) and NF-YB (C-20) were purchased from Santa Cruz Biotechnology (Santa Cruz, CA). Biotin end-labeled or unlabeled oligonucleotides and other chemicals were from Sigma Chemical Co.

### Cell Culture

The human cell line HeLa (cervical cancer, ATCC CCL-2) was maintained in DMEM with 10% FBS. EA.hy926 cells (ATCC CRL-2922), which were derived from fusion of HUVEC with A549 cells (lung adenocarcinoma epithelial cell line, ATCC CCL-185), were cultured in DMEM that contained 10% FBS and HAT media supplement. EA.hy926 cells were a gift from Dr. Cora Edgell at the University of North Carolina.

### Plasmid constructs

The reporter constructs for the exon 1a promoter and the various exon 1a mutants were prepared by PCR with −1983/+266-luc [10] as the template. Digested PCR products were inserted into pGL3-basic vector and verified by sequencing. The expression vectors for NF-Y subunits, YY1, dominant-negative mutant YY1S339/S342 and USF1/2 were all previously described [22], [45], [46], [47]. The expression constructs p300 and CBP were purchased from Addgene (MA).

### Transient transfection and luciferase assays

Cells were seeded in 24-well plates and grown to ∼70% confluence. On the following day, the cells were co-transfected with 0.1 to 0.5 µg of reporter plasmid, 5 ng of *Renilla* plasmid pRL-CMV, 0.3 to 0.9 µg of plasmids expressing the genes of interest or empty vector plasmid to compensate for the amount of DNA. Fugene HD reagent (Roche, IN) was used for the delivery of plasmids into cells. At 24 h after transfection, cell lysates for measurement of firefly and *Renilla* luciferase activities were prepared using Passive Lysis Buffer (Promega) according to the manufacturer's directions.

### Electrophoresis mobility shift assays (EMSA)

For *in vitro* binding reactions, 2 µl of nuclear extract were incubated with the biotin end-labeled probes at room temperature for 20 minutes in 10 mM Tris pH 7.5, 50 mM KCl, 50 mM NaCl, 1 mM DTT, 0.5 mM EDTA, 5% glycerol, 0.1% NP-40, 5 mM MgCl_2_, 1 mg/ml BSA, and 50 ng/µl poly dI-dC. For competition or super-shift experiments, the nuclear extracts were treated with excessive amounts of unlabeled probes or 4 µg antibody for 30 minutes at room temperature prior to the addition of the biotin end-labeled probes. The reaction mixes were then loaded onto 4.5% polyacrylamide gel electrophoresis (PAGE) and run at 100 V in 0.5× TBE buffer for 1.5 hours following the detection according to the manufacturer's directions.

### Co-immunoprecipitation and Western blotting

For co-immunoprecipitation assays, 1×10^7^ HeLa cells were collected, washed in cold-PBS, suspended in 1.5 ml cold lysis buffer, and incubated on ice for 30 min with occasional mixing. The cell lysate was centrifuged at 10,000 g for 10 min at 4°C to pellet cellular debris. The supernatant was treated with 2 µg of antibody and 20 µl of protein G plus-agarose beads (Santa Cruz, CA) for overnight incubation. The bead-antibody pellets were washed with lysis buffer containing 0.5 M NaCl, suspended in 1×SDS loading buffer and boiled for 5 min. Proteins were subjected to polyacrylamide gel electrophoresis on 7.5% polyacrylamide gels and were then electro-transferred to polyvinylidene difluoride membranes. Membranes were blocked with 5% non-fat dry milk for 1 h at room temperature and then incubated for 2 h at room temperature with primary antibodies. After washing, the membranes were incubated at room temperature for 2 hours with a 1∶10,000 dilution of a horseradish peroxidase-conjugated secondary antibody (Sigma) and detection was carried out with ECL (Amersham, UK).

### Chromatin immunoprecipitation assay

Chromatin immunoprecipitation assay (ChIP) assays were performed via a commercially chromatin immunoprecipitation kit (Cell Signaling, MA), using antibodies against either YY1, NF-YA, USF1/2 or p300. Briefly, cell contents were first cross-linked by adding formaldehyde. Cross-linked lysates were then digested by *Micrococcal* nuclease. After digestion, the samples were centrifuged and the supernatants were diluted 5-fold in ChIP buffer. Cross-linked chromatin was incubated overnight with YY1, NF-YA, USF1/2, p300 antibody or normal rabbit IgG at 4°C. Antibody-protein-DNA complexes were isolated by immunoprecipitation with 30 µl of protein G magnetic beads. After extensive washing, pellets were eluted and formaldehyde cross-linking was reversed by 2-h incubation at 65°C after addition of proteinase K and NaCl. Samples were purified and used as a template for PCR. ChIP primers 5-GTT CCA CTT TCA CCC ACG TGA GC-3 and 5-GAG AAG ATG AAT GGC CCT GCT CCA-3 were used to amplify a 119-base pair fragment corresponding to the core exon 1a promoter.

### Lentivirus packaging and knockdown

shRNA sequences were selected according to published criteria [48]: YY1i-AACCTGAAATCTCACATCTTA [49], NFYAi-GCCCTTTACTACAGGACAGAA (Sigma), and TurboGFPi control-CGTGATCTTCACCGACAAGAT (Sigma). Oligos coding for the shRNAs were designed and cloned into the lentiviral vector pLKO.1 [50].

For lentivirus packaging, 50–60% confluent HEK293T cells (ATCC CRL-11268) were transfected using Fugene HD reagent (Roche) with a mixture of 10 µg of pLKO.1 shRNA plasmid, 7.5 µg of envelope encoding pMD2.G plasmid, and 2.5 µg of the packaging vector psPAX2 plasmid (Addgene). The culture supernatant containing the packaged virus was harvested 48 h after transfection and titered p24 concentration (ng/ml) by ELISA. A general guideline is 1 ng p24 = 10^5^ Transducing Units (TU) [51]. Transduction of EA.hy926 cells was performed with recombinant pLKO.1 lentivirus in the presence of 8 µg/ml polybrene as transduction enhancer. Cells were continuously cultured for 4 days before shRNA-mediated knockdown experiments were performed.

### RNA isolation and RT-PCR

Total RNA was isolated using TRIZOL reagent (GIBCO, Grand Island, NY), and 2 µg was reverse-transcribed using iScript cDNA synthesis kit (Bio-Rad, Carlsbad, CA). Amplification was performed using Advantage 2 PCR kit (Clontech, Mountain View, CA). The PCR products were separated on 1% agarose gels. The parameters and primers for SVCT2 exon 1a were described previously [6].

### Data analysis

Statistical significance was determined by analysis of variance with post-hoc testing using the software program Sigma Stat 2.0 (Jandel Scientific, San Rafael, CA). Significance was based on a *P* value of <0.05.
